# Healthcare costs related to adverse events in hepatocellular carcinoma treatment: A retrospective observational claims study

**DOI:** 10.1002/cnr2.1504

**Published:** 2021-09-07

**Authors:** Lincy S. Lal, Abdalla Aly, Lisa B. Le, Susan Peckous, Brian Seal, April Teitelbaum

**Affiliations:** ^1^ Health Economics and Outcomes Research Optum Eden Prairie Minnesota USA; ^2^ US Medical Affairs AstraZeneca Gaithersburg Maryland USA; ^3^ Hematology Oncology Associates San Diego California USA

**Keywords:** adverse events, hepatocellular carcinoma, liver‐directed chemotherapy

## Abstract

**Background:**

Hepatocellular carcinoma (HCC) is an aggressive form of liver cancer with increasing incidence and mortality worldwide. For metastatic disease, systemic treatment is recommended. In addition to tumor characteristics, adverse events (AEs) may influence regimen choice.

**Aim:**

To analyze healthcare burden among patients with advanced HCC, by treatment type and AEs observed.

**Methods:**

Included were adult commercial and Medicare Advantage enrollees with ≥2 non‐diagnostic claims coded for HCC (the first setting the index date); ≥1 claim for systemic treatment of advanced/metastatic HCC; and continuous enrollment for a 6‐month pre‐index baseline period to ≥1 month post‐index (follow‐up). Patients were excluded by lack of systemic treatment; incomplete demographic information; pregnancy, liver transplant, other cancers during baseline or clinical trial participation. We describe patient characteristics, common AEs, overall survival, and healthcare burden in 2017 USD up to 12 months after initiation of tyrosine kinase inhibitor (TKI) monotherapy; immune checkpoint inhibitor (ICI) monotherapy; or FOLFOX combination therapy.

**Results:**

The analytic sample consisted of 322 patients (median age 65.8 years, 76% male) who had 12 months' (unless death occurred prior) available follow‐up, with median follow‐up of 9 months. Among these, 241 (75%) had TKI monotherapy, 23 (7%) had ICI monotherapy, and 58 had FOLFOX (18%) first‐line treatment. Overall, patients had a high burden of AEs (mean 3.2), with the most prevalent being pain (75%), infection (39%), ascites (34%), and bleeding (29%). After adjusting for covariates, infection ($50 374), fever ($47 443), and diarrhea ($29 912) imposed the highest incremental annual costs versus patients without the AE. Up to 90% of costs were attributable to inpatient admissions, with 56% to 60% involving intensive care. Median 1‐year survival was 32%.

**Conclusions:**

This real‐world study demonstrated AE burden in alignment with previous clinical studies. Regardless of regimen used, AEs are associated with substantial healthcare costs due to inpatient care.

## 1 INTRODUCTION

Hepatocellular carcinoma (HCC) is an aggressive tumor that is typically diagnosed at a late stage and most often among patients with chronic liver disease. The presentation is heterogeneous in that patients in the United States (US) are mostly male and have cirrhosis due to alcohol abuse or hepatitis C virus. In contrast, among patients in the Asian‐Pacific regions the underlying disease is more commonly hepatitis B virus infection.^1^, ^2^ HCC accounts for 75–85% of liver cancers and represents a significant cause of cancer‐related mortality.^3^ Despite the association of HCC with modifiable risk factors, incidence and mortality due to HCC continue to rise worldwide.^3^, ^4^ The American Cancer Society estimates that 42 810 new cases of liver cancer (including intrahepatic bile duct cancers) will be diagnosed in the US in 2020, and approximately three‐quarters of those will be HCC.^5^

In instances of early‐stage disease, treatment may include surgical and non‐surgical therapies. Systemic therapies are most often used in advanced‐stage disease.^6^, ^7^, ^8^, ^9^ For metastatic disease, systemic treatments are recommended by the most recent National Comprehensive Cancer Network (NCCN) guidelines.^10^ Unfortunately, underutilization of treatment is common.^2^, ^11^ Indeed, approximately 50% of patients with HCC of any stage do not receive treatment. In addition to performance status and comorbid conditions, demographic factors associated with nontreatment include older age, non‐Caucasian race, and lack of insurance or low socioeconomic status.^11^ Even with treatment for advanced stage disease, US 5‐year survival is 12% or less.^12^ Although tumor characteristics influence the treatment selected, in addition to survival, other important outcomes affecting the choice of regimen include the occurrence of adverse events (AEs) during treatment. Among clinical trials of systemic regimens, common adverse events include hypertension, diarrhea, decreased appetite, nausea/vomiting, myelosuppression, infections, rash, pruritus, and fatigue.^13^, ^14^, ^15^, ^16^, ^17^

Such AEs may lead to reduced survival and quality of life, discontinuation of HCC treatment, and increased costs for healthcare related to treatment of these events.^2^ Randomized clinical trial literature on efficacy and safety exist for systemic treatments of advanced and unresectable HCC. However, data from US real‐world studies have been limited primarily to the study of sorafenib, until recent Food and Drug Administration (FDA) approvals of additional tyrosine kinase inhibitors (TKIs) and immune checkpoint inhibitors (ICIs) added newer options. In this study, NCCN‐recommended treatment regimens for advanced/metastatic and unresectable HCC were studied to describe patient characteristics, the most common AEs during treatment, overall survival, and healthcare costs and utilization for up to 12 months after treatment initiation.

## 2 METHODS

### 2.1 Study design

**FIGURE 1:**
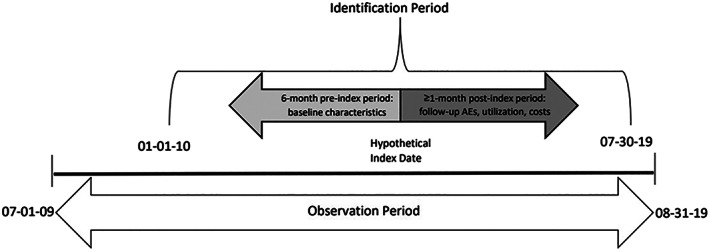
Study design describing a hypothetic index date and pre‐ and post‐index data obtained. AE = adverse event

This was a retrospective database study of costs related to AEs, as observed in administrative healthcare claims, following initiation of treatment of advanced/metastatic HCC. Claims and enrollment information, and linked mortality data, were accessed for the period July 1, 2009 to August 31, 2019 (Figure 1). Baseline patient characteristics (clinical and demographic) were observed during the 6‐month period prior to the index date, which was set as the date of the first claim with an HCC diagnosis during the observation period. The primary outcomes included AEs and healthcare costs and utilization by study cohort assigned by treatment regimen, for the post‐index period of at least 1 month. The sample of patients described herein had at least 12 months of follow‐up available, except in the case of death as end to their study period.

### 2.2 Data sources

Claims data were accessed through the Optum Research Database ([ORD] which is a proprietary database with medical and pharmacy claims data (including linked enrollment) from 1993 to 2020, covering more than 67 million lives. In 2018, approximately 19% of the US commercially enrolled population, and 21% of the Medicare Advantage population (with medical and pharmacy claims) were represented in the ORD. The underlying information is geographically diverse across the United States. Death data were obtained from the Social Security Administration (SSA), Center for Medicare and Medicaid (CMS), and the National Death Index (NDI), in addition to claims‐based sources, such as reason for discharge or disenrollment.

### 2.3 Patient selection/eligibility criteria

The study patients were commercial and Medicare Advantage enrollees with evidence of HCC (at least two non‐diagnostic claims for HCC in any claim position on two separate days during the identification period). Additional inclusion criteria were age ≥ 18 years as of the index date; ≥1 claim for treatment of HCC on or after the index date with NCCN‐recommended systemic regimen for HCC as the first treatment regimen (Appendix 1); continuous health insurance enrollment from 6 months before to ≥1 month after the index date; evidence of advanced/metastatic HCC diagnoses during the 6 months pre‐index, with no evidence of NCCN‐recommended systemic treatments prior to the metastatic date; and complete demographic information.

Exclusion criteria were liver transplant during the 6‐month baseline period; pregnancy during the baseline period; any clinical trial participation at any time during the study period; and ≥ 2 *International Classification of Diseases* (ICD) 9th and 10th Clinical Modifications codes (Appendix 2) ≥30 days apart indicating any other cancer during the baseline period.

Cohorts were assigned by specific NCCN‐recommended regimens of interest only: TKI monotherapy (sorafenib, levatinib, and regorafenib); ICI monotherapy (PD‐1 inhibitor monotherapy, nivolumab, or pembrolizumab); and FOLFOX (fluorouracil, leucovorin, and oxaliplatin or fluorouracil and oxaliplatin) combination therapy.

### 2.4 Study variables

#### 2.4.1 Baseline characteristics

Demographic variables included age as of the index date, gender, insurance type (commercial or Medicare Advantage), and US Census‐designated geographic region.^18^ Clinical comorbid status was indicated by the Charslon^19^, ^20^ Comorbidity Index (CCI) score. In addition, mean and standard deviation (SD) and median time from metastatic diagnosis to start of first line of treatment and follow‐up time were measured in days.

#### 2.4.2 Treatment patterns

The NCCN‐recommended agents for systemic treatment of HCC were identified per the Healthcare Common Procedure Coding System (HCPCS) and an algorithm classified the start of therapy as the date of first claim for a fill or infusion within the first 30 days after the index date. The end of a line of therapy (LOT) was identified by start of a new agent, death, or a 60‐day period of no fills added to a 30‐day run ‐out period, used to identify discontinuation. In addition, a censored (incomplete) LOT was indicated by end of the study period or disenrollment. The proportion of patients who had a second LOT, and the time to the start of LOT2 were recorded.

#### 2.4.3 Outcomes

The AEs of interest were selected through literature and clinical review and package insert reviews for TKIs, ICIs, and FOLFOX, the agents included in NCCN guidelines as of October 2019.^21^, ^22^, ^23^, ^24^, ^25^ Twenty‐five AEs were identified by ICD‐9/10 codes (Appendix 2) appearing as of the first date in which a diagnosis code was observed in the first claim position during the available follow‐up period. All immune‐mediated AEs were reported, as well all other AEs with a prevalence of ≥5% in the in the total sample of patients.

All‐cause healthcare resource utilization was reported as number and proportion of patients having at least one claim for ambulatory (hospital outpatient and physician office), emergency department (ED), inpatient admissions, or intensive care unit (ICU) admissions. Payer‐paid healthcare costs for all‐cause and AE‐related utilization were calculated as per‐patient‐per‐month (PPPM) in $US for ambulatory (hospital outpatient and physician office), ED, inpatient admissions, ICU, other medical, and pharmacy use, adjusted to 2017 USD. The AE‐related costs were based on Claim Status Codes (CSC) indicating AEs only in position #1 on claims to prevent double counting per encounter.

Mortality data (month and year of death) were obtained from the linked SSA Death Master File (DMF), CMS, and the NDI file from the Centers for Disease Control and Prevention (CDC). Death date was identified based on the first date of any of the following: record of death in the SSA DMF; a facility claim with a discharge status field indicating death; a medical claim with a diagnosis code that indicates death; a record of death from CMS for Medicare Advantage patients; discharge and disenrollment reason indicating death; or death dates from NDI file. Death data were used to calculate overall survival for all patients who met the inclusion and exclusion criteria. The period in days between the index date and the end of study was determined for each patient. This allowed determination as to whether the patient died by the end date of the study or was censored at the end of the 12‐month follow‐up period or at disenrollment.

### 2.5 Statistical analyses

All study variables were analyzed descriptively as numbers and proportions (%) for dichotomous and polychotomous variables and means, medians, and SD provided for continuous variables. Descriptive techniques that account for variable length of observation time (e.g., PPPM) were used where appropriate.

Results were stratified by treatment cohort. Bivariate comparisons of pre‐index characteristics and outcome measures were provided, and appropriate tests (e.g., t‐test, Wilcoxon rank‐sum, chi‐square test) were used based on the distribution of the measure. Statistical significance was set at *p* < 0.05. In addition, multivariable analysis of total healthcare costs was conducted using a generalized linear model with log link and gamma distribution, adjusting for the presence of covariates, including the prevalent AEs of at least 5% in the study population. Predicted incremental healthcare costs for patients with versus without select AEs were presented as recycled predictions. Kaplan–Meier (KM) analysis was used to estimate patient overall survival and the fraction of patients surviving for a set period of time after treatment. All analyses were conducted using SAS software version 9.0.

## 3 RESULTS

**FIGURE 2:**
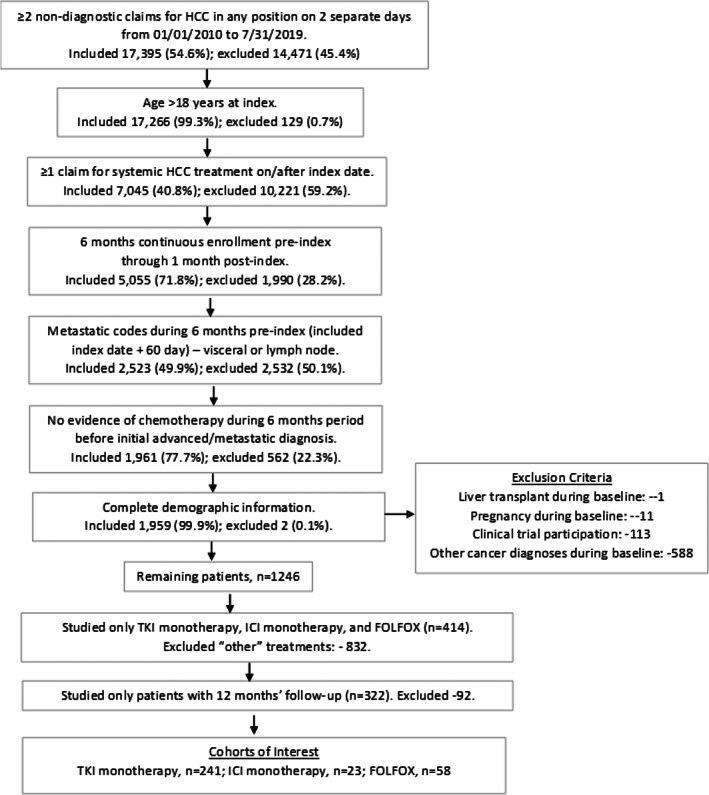
Sample selection and attrition process indicating inclusion and exclusion criteria and steps to the final cohorts of interest. HCC = hepatocellular carcinoma; FOLFOX = fluorouracil, leucovorin, and oxaliplatin or fluorouracil and oxaliplatin; ICI = immune checkpoint inhibitor; TKI = tyrosine kinase inhibitor

After meeting inclusion criteria, 1246 eligible patients were identified (Figure 2). Among them, 414 patients met all inclusion and exclusion criteria, and an analytic sample further limited to only include patients with at least 12 months (unless death occurred prior) of follow‐up and first‐line systemic treatments of interest (total *N* = 322): TKI monotherapy (*n* = 241; 75%); ICI monotherapy (*n* = 23; 7%); and FOLFOX (*n* = 58; 18%).

### 3.1 Sample characteristics

The total analytic sample consisted of 322 patients (median age 65.8 years, 76% male) with 12 months of follow‐up available, except in the event of death before 12 months. The median follow‐up period was 9 months (Table 1). By treatment, 241 (75%) were treated with TKI monotherapy, 23 (7%) with ICI monotherapy, and 58 with FOLFOX (18%), as LOT1. Among all 322 patients, 79 (25%) had ≥2 LOTs. At diagnosis, the mean CCI was 5.1. Geographic distribution aligned with the database overall, with the highest proportion of patients in the South region.

**TABLE 1:** Demographic and clinical characteristics

Characteristics	Total sample (*N* = 322)	TKI Monotherapy (*n* = 241)	ICI Monotherapy (*n* = 23)	FOLFOX (*n* = 58)
Age, mean (SD)	65.8 (10.8)	65.6 (10.2)	73.1 (9.0)	64.1 (12.6)
Proportion female, *n* (%)	76 (23.6)	46 (19.1)	7 (30.4)	23 (39.7)
Coverage type				
Commercial, *n* (%)	126 (39.1)	104 (43.2)	2 (8.7)	20 (34.5)
Younger COM (<65), *n* (%)	104 (82.5)	85 (81.7)	1 (50.0)	18 (90.0)
Older COM (65+), *n* (%)	22 (17.5)	19 (18.3)	1 (50.0)	2 (10.0)
Medicare Advantage, *n* (%)	196 (60.9)	137 (56.8)	21 (91.3)	38 (65.5)
Geographic region				
Northeast, *n* (%)	45 (14.0)	32 (13.3)	3 (13.0)	10 (17.2)
Midwest, *n* (%)	78 (24.2)	54 (22.4)	8 (34.8)	16 (27.6)
South, *n* (%)	155 (48.1)	117 (48.5)	12 (52.2)	26 (44.8)
West, *n* (%)	44 (13.7)	38 (15.8)	0 (0.0)	6 (10.3)
Baseline advanced/metastatic disease^a^, *n* (%)	322 (100.0)	241 (100.0)	23 (100.0)	58 (100.0)
Time from metastasis diagnosis to first‐line treatment (days),^b^ mean (SD)	62 (104)	62 (112)	108 (108)	45 (53)
Follow‐up time, continuous, days, mean (SD)	279 (287)	266 (296)	250 (200)	344 (272)
Baseline Charlson comorbidity score^c^ (continuous), mean (SD)	5.1 (3.6)	5.0 (3.7)	5.2 (3.2)	5.6 (3.2)

*Abbreviations*: COM = commercial; FOLFOX = fluorouracil, leucovorin, and oxaliplatin or fluorouracil and oxaliplatin; ICI = immune checkpoint inhibitor; SD = standard deviation; TKI = tyrosine kinase inhibitor.

^a^ Baseline metastases identified from the index date – 180 through the index date +60.

^b^ Among patients with baseline advanced/metastatic disease and observed first‐line systemic chemotherapy during the follow‐up period. Time from metastatic diagnosis to first‐line treatment = (first‐line therapy starting date) – (metastasis diagnosis date) + 1.

^c^ Quan et al. Updating and validating the Charlson comorbidity index and score for risk adjustment in hospital discharge abstracts using data from 6 countries. *Am J Epidemiology*. 2011; 173(6): 676–682.

### 3.2 Adverse events

Among the analytic sample (*n* = 322) a mean (SD) of 3.2 (2.1) AEs were observed, ranging from 3.1 (2.0) for TKI to 3.6 (2.2) for FOLFOX (Table 2). The most prevalent AEs were pain (75%), infection (39%), ascites (34%), bleeding (29%), and anemia (18%). The top two events for each cohort are as follows: TKI–pain (76%) and infection (39%); ICI–pain (78%) and bleeding (39%); FOLFOX–pain (72%) and infection (47%).

**TABLE 2:** Clinically significant adverse events, by HCC therapeutic regimen

AEs observed during 12‐month follow‐up^c^	Total sample (*N* = 322)	TKI Monotherapy (*n* = 241)	ICI Monotherapy (*n* = 23)	FOLFOX (*n* = 58)
Any AE, *n* (%)	303 (94.1)	225 (93.4)	20 (8.7)	58 (100.0)
Count of AEs, mean (SD)	3.2 (2.1)	3.1 (2.0)	3.3 (2.4)	3.6 (2.2)
Asthenia/fatigue, *n* (%)	52 (16.2)	38 (15.8)	4 (17.4)	10 (17.2)
Bleeding, *n* (%)	94 (29.2)	70 (29.1)	9 (39.1)	15 (25.9)
Thrombocytopenia, *n* (%)	16 (5.0)	9 (3.7)	0 (0.0)	7 (12.1)
Increased AST/ALT, *n* (%)	18 (5.6)	12 (5.0)	2 (8.7)	4 (6.9)
Hyponatremia, *n* (%)	27 (8.4)	23 (9.5)	3 (13.0)	1 (1.7)
Infection, *n* (%)	127 (39.4)	94 (39.0)	6 (26.1)	27 (46.6)
Ascites, *n* (%)	111 (34.5)	89 (36.9)	7 (30.4)	15 (25.9)
Anemia, *n* (%)	59 (18.3)	37 (15.4)	6 (26.1)	16 (27.6)
Diarrhea, *n* (%)	43 (13.4)	31 (12.9)	1 (4.4)	11 (19.0)
Fever, *n* (%)	47 (14.6)	32 (13.3)	5 (21.7)	10 (17.2)
Nausea/vomiting, *n* (%)	56 (17.4)	33 (13.7)	4 (17.4)	19 (32.8)
Pain, *n* (%)	242 (75.2)	182 (75.5)	18 (78.3)	42 (72.4)
Any immune‐mediated (IM) AE, *n* (%)	69 (21.4)	52 (21.6)	7 (30.4)	10 (17.2)
IM hepatitis, *n* (%)^a^	28 (8.7)	20 (8.3)	4 (17.4)	4 (6.9)
IM colitis, *n* (%)	13 (4.0)	8 (3.3)	0 (0)	5 (8.6)
IM diabetes, *n* (%)^b^	8 (2.5)	7 (2.9)	1 (4.4)	0 (0)
IM hypothyroidism, *n* (%)	14 (4.4)	12 (5.0)	0 (0)	2 (3.5)
IM hyperthyroidism, *n* (%)	1 (0.3)	0 (0)	0 (0)	1 (1.7)
IM cardiomyopathy/myocarditis, *n* (%)	4 (1.2)	1 (0.4)	2 (8.7)	1 (1.7)
IM pancreatitis, *n* (%)	13 (4.0)	11 (4.6)	1 (4.4)	1 (1.7)
IM thrombocytopenia, *n* (%)	1 (0.3)	1 (0.4)	0 (0)	0 (0)
IM inflammatory arthritis, *n* (%)	1 (0.3)	1 (0.4)	0 (0)	0 (0)

*Note*: Immune‐mediated nephritis, pneumonitis, pituitary/adrenal insufficiency, red cell aplasia/hemolytic anemia/rhabdomyolysis, and cutaneous dermatitis/Stevens‐Johnson syndrome or toxic epidermal necrolysis were also examined and were observed in none of the patients' claims during the observation period. *Abbreviations*: AST = aspartate aminotransferase; ALT = alanine aminotransferase; AE = adverse event; FOLFOX = fluorouracil, leucovorin, and oxaliplatin or fluorouracil and oxaliplatin; HCC = hepatocellular carcinoma; ICI = immune checkpoint inhibitor; IM = immune mediated; SD = standard deviation; TKI = tyrosine kinase inhibitor.

^a^ No increase in AST/ALT was used in the definition of IM hepatitis.

^b^ Type 1 diabetes occurring during ICI therapy was considered immune‐mediated.

^c^ Except for immune‐mediated conditions, categories shown include only those with prevalence of at least 5% of total sample.

### 3.3 Outcomes

All‐cause utilization by regimen during follow‐up among the analytic sample (*n* = 322) was high for ED and inpatient hospitalizations. In total, 84% of patients had ≥1 ED visit, 81% had ≥1 inpatient hospitalization, and among inpatients, 46% had ≥1 ICU stay. The values for ED, inpatient, and ICU all‐cause utilization for those receiving TKI monotherapy (*n* = 241) were 82%, 81%, and 45%, respectively. Among those receiving ICI monotherapy (*n* = 23) were 91%, 83%, and 47%, respectively, and for FOLFOX (*n* = 58) were 90%, 83%, and 50%, respectively.

**FIGURE 3:**
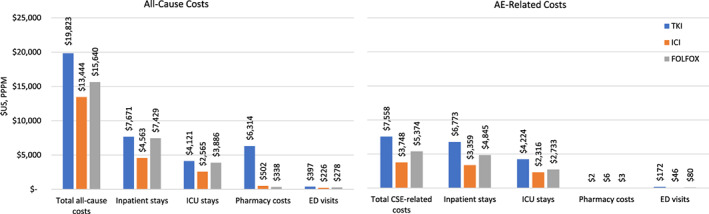
All‐cause and AE‐related healthcare costs in $US for each cohort of interest, by category of cost type. AE = adverse event; ER = emergency room; FOLFOX = fluorouracil, leucovorin, and oxaliplatin or fluorouracil and oxaliplatin; ICI = immune checkpoint inhibitor; ICU = intensive care unit; PPPM = Per patient per month; TKI = tyrosine kinase inhibitor; US=United States

Payer‐paid all‐cause PPPM costs by category and first‐line treatment are shown in Figure 3(A) for the analytic sample (*n* = 322). Inpatient hospitalization incurred approximately one third to one half of the total costs among all treatments. As a fraction of inpatient costs, 54%, 56%, and 52% of costs were for ICU care for TKI monotherapy, ICI monotherapy, and FOLFOX cohorts, respectively. The majority (90%) of total AE‐related costs, regardless of treatment regimen, were attributable to inpatient care. Among the inpatient AE‐related healthcare costs, 62%, 69%, and 56% were for ICU care, among the TKI monotherapy, ICI monotherapy, and FOLFOX cohorts, respectively. Patients receiving TKI monotherapy had the highest PPPM all‐cause costs and AE‐related costs, driven mostly by inpatient costs. AE‐related costs were similar when the 19 patients without AEs were excluded for a sample of *n* = 303 patients (data not shown).

**FIGURE 4:**
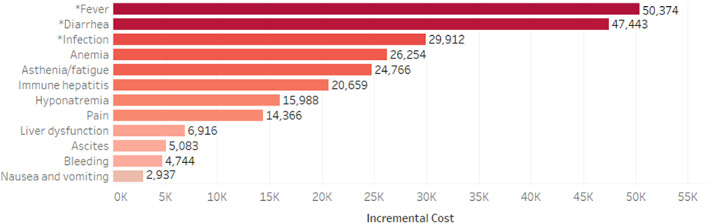
Predicted incremental annual all‐cause healthcare costs in $US associated with adverse events. *Statistically significant incremental costs over 12‐month follow‐up period among patients with 12 months of follow‐up available (less in the event of death). AE = adverse event

Figure 4 shows incremental costs over the total follow‐up period associated with specific AEs, as determined by multivariable analysis of total all‐cause healthcare costs, adjusting for important covariates and LOT1 treatment regimen. Only the AEs with ≥5% prevalence were included in the final model. AEs associated with statistically significant differences in incremental costs included infection, fever, and diarrhea, at $50 374, $47 443, and $29 912, respectively. See Appendix 3 for full list of covariates, cost ratios, and confidence intervals.

The survival rate was 32% for the total population (*N* = 414) at 1‐year follow‐up, with median survival of 6.97 months (95% confidence intervals [CI]: 6.33–7.90) for TKI versus 7.23 months (95%CI:5.47–13.67) for ICI monotherapy, versus 10.37 months (95%CI: 7.43–14.43) for the FOLFOX cohort.

## 4 DISCUSSION

This study is one of few studies based on real‐world samples; most studies present highly selected RCT populations, which have homogeneous characteristics by design. Recent systematic reviews,^2^, ^26^ including studies not necessarily designed to examine costs associated with adverse events, have supported the conclusion that HCC imposes a substantial burden upon patients, caregivers, and the healthcare system. The current analytic sample included 322 patients with 12 months of follow‐up available, except for those whose study period ended earlier due to death, but not due to discontinuation, end of enrollment, or end of the study. The majority of patients (75%) received TKI, with 7% receiving ICI, and 18% FOLFOX. With a similar proportion of patients receiving one line of sorafenib treatment, only one recent real‐world study presents a comparable US‐based source.^27^

The demographic characteristics of the current sample align well with those of real‐world populations of patients with HCC previously reported.^2^, ^27^ The US geographic regions most represented were the South and Midwest regions, based upon the distribution of patients in the database, rather than the distribution of cases with HCC overall. Over the period from 2009 to 2019, the mean duration of treatment in LOT1 was 89 days, and 24% of the patients moved on to a LOT2. In a meta‐analysis that reviewed papers published during the period 2011–2018, the mean duration of LOT1 was 60 days and 16% moved on to LOT2.^2^ Another study (period of 2008 to 2015) showed LOT1 of 150 days with 11% of patients receiving LOT2.^27^ It may be as efforts are made to improve treatments used after LOT1, greater numbers of patients pursue a second‐line therapy. However, a large proportion of patients with HCC do not pursue systemic treatment at all; ^2^ this may be associated with advanced disease stage, high burden of comorbid conditions/intolerability,^27^ or sociodemographic reasons.^11^

The most prevalent AEs during follow‐up were pain (75%), infection (39%), ascites (34%), bleeding (29%), and anemia (18%). The rates of TKI‐associated AEs are in line with the previous literature, for which adverse event rates have ranged from 45% for sorafenib to 75% for levatinib,^23^ although rates for individual conditions vary by study, most likely based upon their definitions used. The rates associated with ICI monotherapy for individual events are in line with the literature; however, the total number of any events appear to be higher than previously reported,^28^ although the period of follow‐up also varied between studies. Unfortunately, few studies have been designed specifically to describe AEs in real‐world clinical treatment of patients with HCC, and none to‐date describe incremental costs associated with those AEs. Such data are among several factors useful in determining the appropriate choice of treatment for any individual patient.

The majority of patients observed in this study experienced substantial healthcare utilization and costs, which is consistent with previous reports.^2^, ^26^, ^27^, ^29^ The total all‐cause costs included a greater pharmacy component for patients who received TKI monotherapy than the other regimens studied, as these were all oral agents. Patients who received TKI monotherapy had the highest costs, driven mostly by inpatient care. In fact, the single category with highest costs was inpatient care, across all regimens. Furthermore, the majority (90%) of AE‐related costs, regardless of treatment regimen, were attributable to inpatient care. In addition, among the TKI monotherapy, ICI monotherapy, and FOLFOX cohorts, the proportion of inpatient costs attributable to ICU stays was 62%, 69%, and 56%, respectively. This indicates a substantial burden in healthcare associated with the specific care of AEs during treatment for HCC.

Multivariable analysis indicated that fever, diarrhea, and infection increased the overall total cost significantly in patients with these events, as compared with patients without these events. Fever increases the incremental annual cost by $50 000, diarrhea by $47 000, and infection by $30 000. These AEs incurred higher incremental costs over patients without them, despite their being interrelated conditions. As one example, fever and infection or liver dysfunction or immune hepatitis are likely to co‐occur, and both codes would be present on claims as triggering diagnostic tests and inpatient care. Nevertheless, AEs were identified by codes in the primary position on a claim to avoid double counting of AEs and costs, and only AEs with a prevalence of ≥5% among all patients was included in the model. This study indicates that the PPPM costs for patients with HCC systemic treatment have increased relative to previous studies, as newer agents are being added to the treatment armamentarium.^2^ Identifying costs for care associated with AEs represents a new addition to the literature related to HCC treatment management.

### 4.1 Limitations

Caution is required when interpreting results of comparative observational studies, given the lack of randomization and subsequent biases (e.g., channeling) introduced in an observational design. The use of claims for outcomes studies is limited in that such data are created for the purpose of billing, rather than research, and can only reflect costs paid for care in covered venues/providers and are subject to coding errors. Additionally, claims cannot provide all clinical data, such as grade and severity, which would be useful for comparing AEs in the context of this study relative to those described in randomized controlled trials. As such, it must also be acknowledged that while some AEs are referred to as “immune‐mediated,” there is no way to absolutely confirm this for many of them, as for example in hypothyroidism. In addition, some of the AEs—in particular, pain, ascites, and asthenia/fatigue—could have been due to the HCC itself and possibly exacerbated or not strictly associated with the treatment, but there is no way to determine this. The use of claims also limits the interpretation to patients included with the type(s) of insurance coverage included in the database; findings may not be generalizable to uninsured patients, those with different types of insurance coverage, or those outside of the United States. Furthermore, the results are applicable only to patients with the systemic treatments described herein; they do not represent the experiences of patients receiving other systemic regimens or treatments administered as part of a clinical trial. The data used to perform this study are not publicly available; therefore, inability for others to replicate results may be considered a limitation of the data source. Finally, death data sources are limited, but the use of multiple sources, including linking to NDI, reduces the impact of missing deaths on survival rates.

## 5 CONCLUSIONS

This study suggests that patients with advanced/metastatic HCC incur significant clinical and economic burden in the first year after diagnosis in the US, irrespective of the systemic therapy used. AEs account for a significant portion of health care resource utilization and total costs.

## CONFLICT OF INTEREST

LSL, LBL, and SP were employed by Health Economics and Outcomes Research, Optum, Eden Prairie, MN; AA and BS were employed by US Medical Affairs, AstraZeneca, Gaithersburg, MD; and AT was affiliated with Hematology Oncology Associates, Carlsbad, CA, at the time this work was conducted. AA holds stock in AstraZeneca. No employment was contingent upon publication of this work. Authors declare there are no other conflicts of interest.

## AUTHOR CONTRIBUTIONS

**Lincy Lal:** Conceptualization; data curation; formal analysis; investigation; methodology; project administration; validation; writing‐original draft; writing‐review & editing. **Abdalla Aly:** Conceptualization; formal analysis; investigation; methodology; project administration; validation; writing‐original draft; writing‐review & editing. **Lisa Le:** Data curation; formal analysis; investigation; methodology; writing‐original draft. **Susan Peckous:** Conceptualization; data curation; formal analysis; investigation; project administration; validation; writing‐original draft; writing‐review & editing. **Brian Seal:** Conceptualization; investigation; methodology; project administration; supervision; writing‐original draft; writing‐review & editing. **April Teitelbaum:** Conceptualization; formal analysis; investigation; methodology; validation; writing‐original draft; writing‐review & editing.

## ETHICAL STATEMENT

Institutional Review Board review and approval was obtained. A HIPAA waiver was received specific to the use of identifiable health information.

## Supporting information


**Appendix 1.** Supporting Information.

**Appendix 2.** Supporting Information.

**Appendix 3.** Supporting Information.

## Data Availability

Third party use of the proprietary data obtained from the Optum Research Database requires strict data security and privacy protocols and a restrictive license agreement. Thus, data used to generate the results presented cannot be disclosed publicly.
