# Supplementary material for: Healthcare costs related to adverse events in hepatocellular carcinoma treatment: A retrospective observational claims study
Source: Cancer Rep (Hoboken). 2021 Sep 7;5(5):e1504. doi: 10.1002/cnr2.1504 (PMC9124510; doi:10.1002/cnr2.1504)
Supplement: Supplementary file 3 — Appendix 3. Supporting Information. [file CNR2-5-e1504-s001.docx]

Appendix 3: Multivariable Model of Total All-Cause Health Care Costs during the 12-Month Follow-up Period among Systemic Chemotherapy Patients with 12 Months of Follow-up [or Less if Due to Death]

| **Independent Variables** | **Total all-cause health care costs** | | | | |
| --- | --- | --- | --- | --- | --- |
|  | **cost ratio** | **lower 95% CI** | **upper 95% CI** | **p-value** | **predicted value** |
| Intercept | – | – | – | <0.001 | – |
| **Patient characteristics** |  |  |  |  |  |
| Age | 0.983 | 0.974 | 0.993 | <0.001 | – |
| Gender |  |  |  |  |  |
| Female | 1.028 | 0.846 | 1.250 | 0.778 | – |
| Male | ref. | – | – | – | – |
| Coverage type |  |  |  | <0.001 |  |
| Commercial | 1.515 | 1.221 | 1.880 | <0.001 | – |
| Medicare Advantage | ref. | – | – | – | – |
| Region |  |  |  | 0.414 |  |
| Northeast | 1.230 | 0.960 | 1.576 | 0.102 | – |
| Midwest | 1.082 | 0.885 | 1.323 | 0.443 | – |
| South | ref. | – | – | – | – |
| West | 1.021 | 0.794 | 1.312 | 0.870 | – |
| Baseline Charlson comorbidity score category |  |  |  | 0.381 |  |
| 0 | ref. | – | – | – | – |
| 1-2 | 1.279 | 0.967 | 1.692 | 0.084 | – |
| 3-4 | 1.171 | 0.877 | 1.562 | 0.284 | – |
| 5+ | 1.174 | 0.927 | 1.487 | 0.184 | – |
| **Clinically significant adverse events (AEs)** |  |  |  |  |  |
| Pain |  |  |  |  |  |
| With AE | 1.144 | 0.950 | 1.377 | 0.156 | 114,205.464 |
| Without AE | ref. | – | – | – | 99,839.036 |
| Infection |  |  |  |  |  |
| With AE | 1.305 | 1.100 | 1.548 | 0.002 | 128,041.290 |
| Without AE | ref. | – | – | – | 98,129.529 |
| Anemia |  |  |  |  |  |
| With AE | 1.247 | 0.996 | 1.562 | 0.054 | 132,486.660 |
| Without AE | ref. | – | – | – | 106,232.754 |
| Ascites |  |  |  |  |  |
| With AE | 1.047 | 0.871 | 1.258 | 0.626 | 113,926.393 |
| Without AE | ref. | – | – | – | 108,843.023 |
| Asthenia/fatigue |  |  |  |  |  |
| With AE | 1.232 | 0.984 | 1.542 | 0.068 | 131,434.820 |
| Without AE | ref. | – | – | – | 106,669.287 |
| Nausea and vomiting |  |  |  |  |  |
| With AE | 1.027 | 0.821 | 1.284 | 0.818 | 112,951.938 |
| Without AE | ref. | – | – | – | 110,015.329 |
| Bleeding |  |  |  |  |  |
| With AE | 1.043 | 0.861 | 1.264 | 0.664 | 113,955.529 |
| Without AE | ref. | – | – | – | 109,211.564 |
| Hyponatremia |  |  |  |  |  |
| With AE | 1.146 | 0.831 | 1.581 | 0.406 | 125,366.084 |
| Without AE | ref. | – | – | – | 109,377.857 |
| Fever |  |  |  |  |  |
| With AE | 1.488 | 1.162 | 1.905 | 0.002 | 153,651.474 |
| Without AE | ref. | – | – | – | 103,277.402 |
| Diarrhea |  |  |  |  |  |
| With AE | 1.454 | 1.125 | 1.880 | 0.004 | 151,886.562 |
| Without AE | ref. | – | – | – | 104,443.288 |
| Liver dysfunction |  |  |  |  |  |
| With AE | 1.063 | 0.719 | 1.570 | 0.760 | 117,185.849 |
| Without AE | ref. | – | – | – | 110,270.147 |
| Immune hepatitis |  |  |  |  |  |
| With AE | 1.190 | 0.868 | 1.630 | 0.280 | 129,663.156 |
| Without AE | ref. | – | – | – | 109,004.079 |
| Systemic cancer therapy agents in first line (as of 12-month follow-up) |  |  |  | 0.426 |  |
| TKI monotherapy | ref. | – | – | – | – |
| ICI monotherapy | 1.127 | 0.820 | 1.549 | 0.460 | – |
| Other systemic combination – FOLFOX | 0.897 | 0.718 | 1.121 | 0.339 | – |

*Note*. CI=confidence interval; AE=adverse event; FOLFOX= fluorouracil, leucovorin, and oxaliplatin or fluorouracil and oxaliplatin; ICI=immune checkpoint inhibitor; TKI=tyrosine kinase inhibitor
